# Does size matter? Separations on guard columns for fast sample analysis applied to bioenergy research

**DOI:** 10.1186/s12896-015-0159-3

**Published:** 2015-05-28

**Authors:** Stefan Bauer, Ana B. Ibáñez

**Affiliations:** Energy Biosciences Institute, University of California, Berkeley, CA 94720 USA

**Keywords:** Ion exclusion chromatography, Aminex HPX-87H, Rezex RFQ, Micro Guard Cation H, Enzymatic digestibility assay, Glucose, Ethanol, Butanol, Furans, Fermentation

## Abstract

**Background:**

Increasing sample throughput is needed when large numbers of samples have to be processed. In chromatography, one strategy is to reduce column length for decreased analysis time. Therefore, the feasibility of analyzing samples simply on a guard column was explored using refractive index and ultraviolet detection. Results from the guard columns were compared to the analyses using the standard 300 mm Aminex HPX-87H column which is widely applied to the analysis of samples from many biotechnology- and bioenergy-related experiments such as biomass conversions or fermentations.

**Results:**

The 50 mm Rezex RFQ Fast Acid H^+^ guard column was able to separate the most common fermentation products (ethanol, acetone, iso- and n-butanol) and promising precursors (furfural and 5-hydroxymethylfurfural) of biofuels and value-added chemicals. Compound profiles in fermentation samples were analyzed with similar accuracy compared to results using the 300 mm column. However, separation of glucose and xylose was not achieved. Nevertheless, it was possible to monitor the consumption of one of the two sugars during fermentation if the other one was absent or remained constant over the course of the experiment. If correct peak integration and interference subtraction was applied, concentration profiles from enzymatic digestibility experiments and even more complex samples (e.g. acetone-butanol-ethanol (ABE) fermentation) were reliably obtained. With the 50 mm guard column, samples were analyzed up to ten-times faster compared to the 300 mm column. A further decrease in analysis time was achieved by using the 30 mm Micro Guard Cation H guard column. This column is especially suitable for the rapid analysis of compounds with long elution times on the standard 300 mm column, such as biofuel-related alcohols (e.g., n-butanol, n-hexanol) and furan- and tetrahydrofuran-type molecules.

**Conclusion:**

Applied to a suitable set of samples, separations on a guard column can give rapid and sufficiently accurate information on compound changes over the course of an experiment. Therefore, it is an inexpensive and ideal tool for processing a large amount of samples, such as in screening or discovery experiments, where detecting relative changes is often sufficient to identify promising candidates for further analysis.

## Background

A popular liquid chromatography column used for the analysis of biomass conversion and fermentation products is based on a polymer matrix of polystyrene-divinylbenzene (e.g. Bio-Rad Aminex HPX-87H, Phenomenex Rezex ROA and RFQ, Shodex SH1821, Sigma-Aldrich Supelcogel C610H and others) [1–6]. It is operated in isocratic elution mode enabling the connection to a refractive index detector (RID) which provides universal compound detection. This set up allows for analysis of a wide range of compounds. With a standard 300 mm length, this type of column provides baseline separation of the main cell wall sugars (glucose, xylose and arabinose) of many lignocellulosic biomass feedstocks. This column is also used for the analysis of organic acids, alcohols (e.g., ethanol, n-butanol) and sugar degradation products (e.g., 5-hydroxymethylfurfural, furfural) [7–10]. It only requires acidified water as mobile phase and only minimal sample preparation.

Typical HPLC methods aim to separate complex mixtures in order to individually detect isolated compounds – particularly when a non-specific detector like RID is used. In our experience from supporting researchers in an organization comprising more than 500 scientists dedicated to bioenergy research, many analytical questions can be reduced to identifying the change in a few compounds over the course of the experiment. Furthermore, reducing analysis time is often more important than obtaining precise absolute quantification data. This is especially true for the results from screening experiments involving a larger amount of samples. The detection of relative changes is often sufficient to identify promising candidates for further analysis.

Fast methods targeting individual compounds in complex mixtures have previously been established and include, for example, gas chromatography [11] spectroscopy [12], or enzymatic assays, such as for sugars, sugar alcohols, organic acids, and ethanol [13–17]. Several of these target analytes can even be measured in an automated mode using a biochemistry analyzer (YSI) and results are obtained in minutes [18]. However, all such methods either require costly equipment or individual analysis kits for every single analyte. Therefore, it is tempting to use existing analytical instrumentation (HPLC) and reduce analysis time for higher sample throughput. In this respect, Scarlata and Hyman [6] have successfully shown that reducing the column length to 100 mm (plus a 30 mm guard column) can reduce the analysis time by a factor of five compared to the 300 mm column, but still provide adequate accurate results from a biomass compositional analysis (glucose, xylose, arabinose, acetate, 5-hydroxymethylfurfural, furfural). In our studies, we explored a further reduction of the analysis time by using only a guard column as the simplest and most basic column available. For this purpose, samples generated by various research groups in our institute were analyzed on the standard 300 mm Aminex HPX-87H column and compared to results obtained from an analysis on a guard column (50 mm Rezex RFQ Fast Acid H^+^ as well as 30 mm Micro Guard Cation H).

## Results and discussion

A short column length is often used for faster separation especially in conjunction with mass spectrometric detection. These shorter columns have usually smaller particle sizes (3.5 μm and lower) which greatly improve resolution. However, columns like the Aminex HPX-87H operate in a mixture of size- and ion-exchange/exclusion. They consist of a resin with a rather large particle size (8–9 μm). As a result, a reduction in column length significantly lowers column efficiency. For example, a reduction of the column length to 50 mm results in practically unresolved glucose and xylose (see peak 1 and 2 in Fig. 6a). These types of columns can therefore not be directly compared to other columns. Despite the lower separation efficiency, the shorter columns can still provide adequate chromatographic resolution when used with appropriate samples in which compounds co-eluting with the target analytes are either not present or their concentration is sufficiently low so they do not significantly interfere with quantification.

**Fig. 1:**
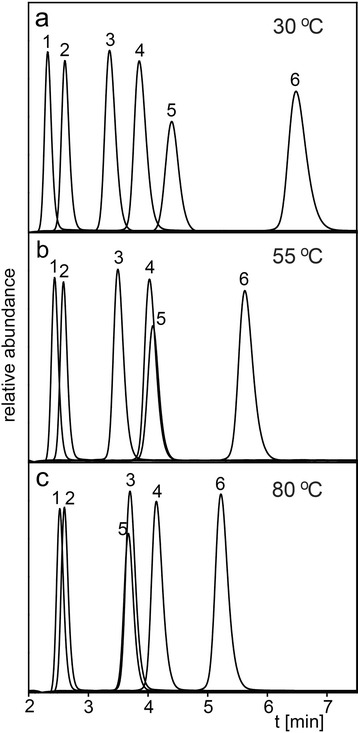
Separation of ethanol (peak 1), acetone (peak 2), iso-butanol (peak 3), n-butanol (peak 4), 5-HMF (peak 5) and furfural (peak 6) on the 50 mm guard column at a column temperature of (**a**) 30 °C, (**b**) 55 °C and (**c**) 80 °C.HPLC parameters: 50 mm × 7.8 mm RFQ Fast Acid H^+^ column, mobile phase 5 mM sulfuric acid, flow rate 1.0 mL/min, refractive index detection

Figure 1 shows the elution profile of some of the most common fermentation products (ethanol, acetone, iso-butanol, n-butanol) and sugar degradation products (5-hydroxymethylfurfural (5-HMF), furfural) on the 50 mm Rezex RFQ Fast Acid H^+^ guard column (“50 mm guard column”) operated at different temperatures at a flow rate of 1.0 mL/min. The degradation products 5-HMF and furfural are promising precursors for the generation of biofuels and value-added chemicals [3, 19, 20]. Good separation of all these compounds is achieved at a column temperature of 30 °C (Fig. 1a). At this temperature, ethanol (peak 1) and acetone (peak 2) are almost baseline separated. By increasing the column temperature the alcohols elute later and 5-HMF (peak 5) and furfural (peak 6) elute earlier. The retention time of acetone (peak 2) is practically not affected by temperature (Fig. 1a-c). Ethanol/acetone (peak 1/peak 2) separation is deteriorated at a higher temperature and 5-HMF (peak 5) is co-eluting with n-butanol (peak 4) at 55 °C (Fig. 1b) and with iso-butanol (peak 3) at 80 °C (Fig. 1c). The column temperature must therefore be chosen based on the sample composition so that target analytes do not overlap. For example, as mentioned above, a temperature of 30 °C has to be applied for successful separation of ethanol and acetone. This column temperature is also beneficial for the faster elution of iso-and n-butanol (Fig. 1a). In contrast, 5-HMF and furfural are eluted faster at a higher temperature. However, depending on which butanol isomer is present, the column temperature needs to be correctly chosen since 5-HMF coelutes either with n-butanol at 55 °C (Fig. 1b) or iso-butanol at 80 °C (Fig. 1c).

Using a 20 μL injection volume, the determined linear calibrations for the compounds under study ranged from 0.005-0.02 mg/mL to 50 mg/mL (20 mg/mL for glucose and xylose) with RI and UV detection (Table 1). UV detection (280 nm) resulted in an about 100-fold increased sensitivity for 5-HMF and furfural compared to RI detection (calibration range 50 ng/mL – 0.25 mg/mL).

**Table 1:** Calibration results for each compound

Compound	Detection	Linear quantification range	Regression coefficient
Ethanol	RID	0.01 − 50 mg/mL	0.9995
Acetone	RID	0.01 − 50 mg/mL	0.9996
	UV 265 nm	0.01 − 50 mg/mL	0.9999
	UV 285 nm	0.02 − 50 mg/mL	0.9995
iso-Butanol	RID	0.005 − 50 mg/mL	0.9999
n-Butanol	RID	0.005 − 50 mg/mL	0.9997
5-HMF	RID	0.005 − 50 mg/mL	0.9999
	UV 280 nm	0.00005 − 0.25 mg/mL	0.9999
Furfural	RID	0.005 − 50 mg/mL	0.9995
	UV 280 nm	0.00005 − 0.25 mg/mL	0.9999
Glucose	RID	0.005 − 20 mg/mL	0.9999
Xylose	RID	0.005 − 20 mg/mL	0.9999

Chromatography conditions: 50 mm × 7.8 mm RFQ Fast Acid H^+^ kept at 30 °C, flow rate: 1.0 ml/min of 5 mM sulfuric acid, 20 μL injection volume; Lower limit of the calibration range was determined as the concentration level with a relative standard deviation (RSD) <10 % (n = 5)

Since 80 °C is close to the maximum operating temperature of the column (85 °C), we chose a column temperature of 55 °C for most of the following experiments. This is good balance between fast analysis time and column life time.

### Application to analysis of n-butanol and iso-butanol production during fermentations

**Fig. 2:**
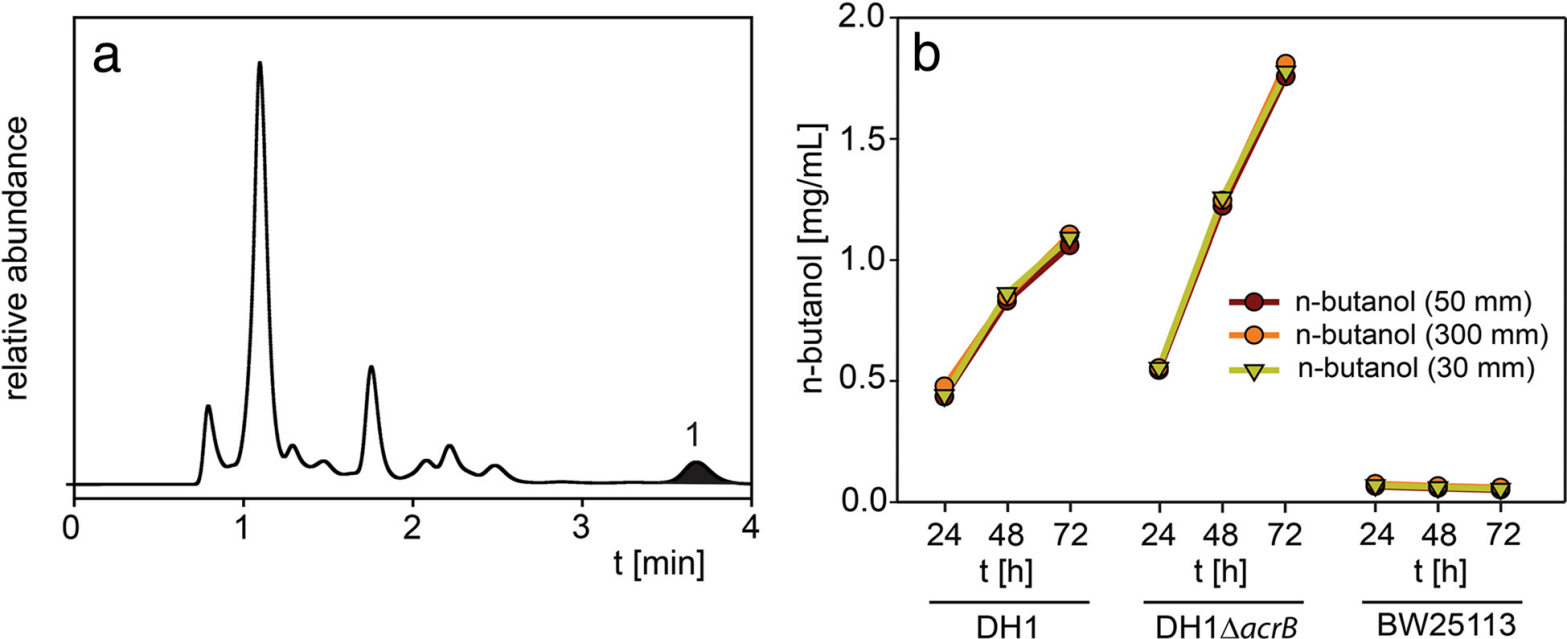
Representative chromatogram of the supernatant from *Escherichia coli* strain DH1 fermentation analyzed with (**a**) the 50 mm guard column showing n-butanol (peak 1) elution, and (**b**) n-butanol concentration of the supernatant from *E. coli* strains DH1, DH1Δ*acrB* and BW25113 fermentations after 24 h, 48 h and 72 h analyzed with the 50 mm guard column, 300 mm column and 30 mm guard column. HPLC parameters: column temperature 55 °C, mobile phase 5 mM sulfuric acid, flow rate 1.0 mL/min (0.6 mL/min for 300 mm column), refractive index detection

These two alcohols elute later than most other components present in the fermentation media. Fig. 2a shows a representative chromatogram from an *E.coli* fermentation producing n-butanol (peak 1) which eluted at 3.7 min. Fig. 2b shows the determined n-butanol production of three *E. coli* strains after incubation for 24 h, 48 h and 72 h. The n-butanol profile obtained with the 50 mm guard column was very similar compared to the 300 mm Bio-Rad HPX-87H column (“300 mm column”). Values reached 90.9-98.0 % of the values obtained with the longer column (except for the BW25113 values which reached only 80.7-84.1 % due to the low concentration affecting accuracy). The DH1 strain is able to produce more n-butanol than BW25113, which almost showed no significant n-butanol levels. Usually, BW25113 produces about half the concentration of DH1 (Niwen Kong, personal communication). Unexpectedly, the BW25113 strain used in this experiment did not perform as expected. The deletion of AcrB efflux pumps in DH1Δ*acrB* lead to even higher n-butanol levels. AcrB pumps are large proteins and are used to actively secret n-butanol from the cell. However, they can be considered “energy drainers” since they use a big portion of the cellular resources needed for n-butanol synthesis. At the levels measured here, n-butanol is not toxic for the *E. coli* cells and therefore the AcrB pumps present in the DH1 strain are not a “de-toxification”-advantage over the DH1*ΔacrB* strain which has more energy resources available for n-butanol production.

**Fig. 3:**
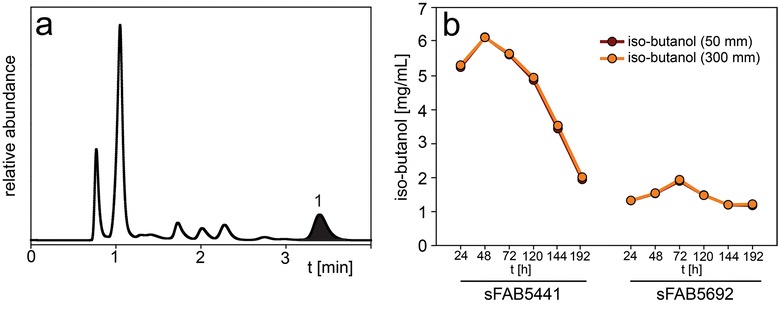
Representative chromatogram of the supernatant from *Escherichia coli* strain sFAB5692 fermentation analyzed with (**a**) the 50 mm guard column showing iso-butanol (peak 1) elution, and (**b**) iso-butanol concentration profile of the supernatant from *E. coli* strains sFAB5441 and sFAB5692 fermentation between 24 h and 192 h analyzed with the 50 mm guard column and 300 mm column. HPLC parameters: column temperature 55 °C, mobile phase 5 mM sulfuric acid, flow rate 1.0 mL/min (0.6 mL/min for 300 mm column), refractive index detection

A similar chromatogram was obtained for iso-butanol production from *E. coli* strains sFAB5441 and sFAB5692 (Fig. 3a). Iso-butanol (peak 1) eluted at 3.4 min in a region where no interference from other media components were expected. The values measured with the 50 mm guard column were again almost identical to the ones obtained with the 300 mm column (in the range of 96.7-100.4 %, Fig. 3b). In this experiment, the decrease of iso-butanol after 48 h for the sFAB 5441 strain was explained by evaporation due to a leak in the cap of the flask.

The analysis of n- and iso-butanol was performed with a 4 min total run time and this is about 9–10 times faster compared to the standard 300 mm column.

### Application to analysis of 5-hydroxymethylfurfural (5-HMF) and furfural

**Fig. 4:**
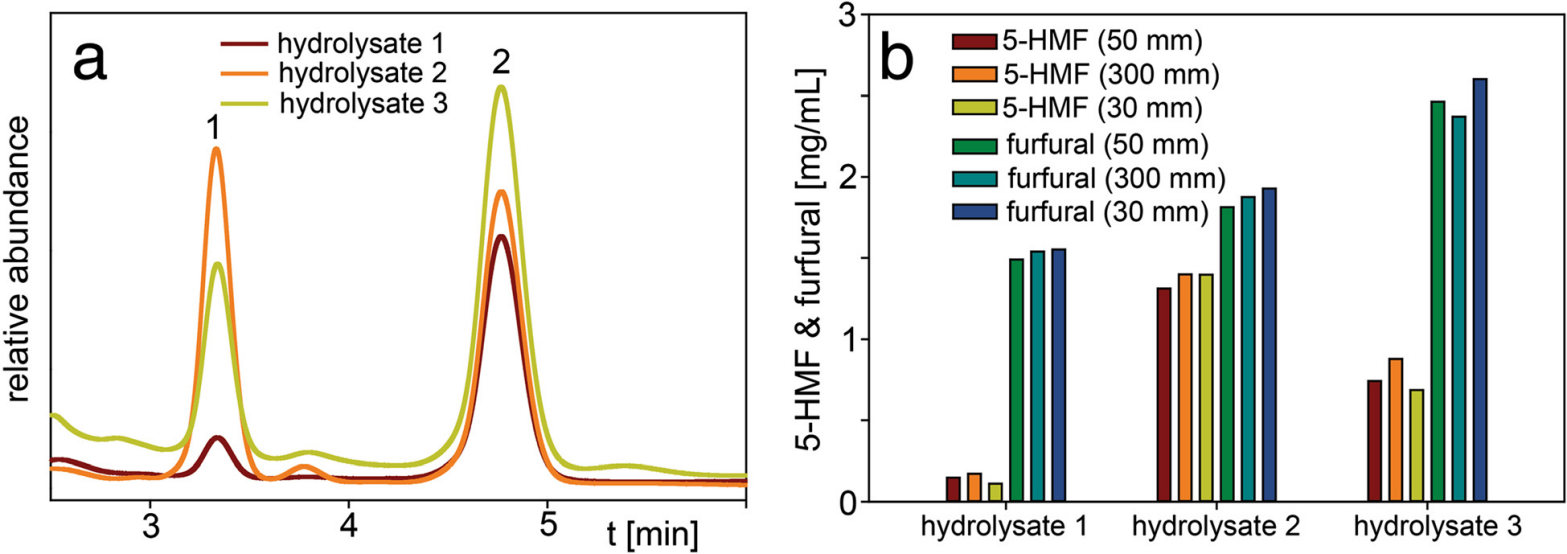
Chromatogram of hydrolysate 1, hydrolysate 2 and hydrolysate 3 analyzed with (**a**) the 50 mm guard column showing 5-HMF (peak 1) and furfural (peak 2) elution, and (**b**) 5-HMF and furfural concentrations of hydrolysate 1, hydrolysate 2 and hydrolysate 3 analyzed with the 50 mm guard column, 300 mm column and 30 mm guard column. HPLC parameters: column temperature 55 °C, mobile phase 5 mM sulfuric acid, flow rate 1.0 mL/min (0.6 mL/min for 300 mm column), refractive index detection

5-HMF and especially furfural are compounds with long elution times on the standard 300 mm HPX-87H column (at a flow rate of 0.6 mL/min and 50 °C about 34 and 52 min, respectively). Experiments requiring the analysis of these compounds often include determination of the degree of their removal or reduction during membrane detoxification of hydrolysate [21] or the rate of sugar degradation during pretreatment or conversion steps [9, 10]. For the purpose of this study, three biomass hydrolysates prepared under different severity conditions were analyzed. Fig. 4a depicts the elution profiles for the two compounds on the 50 mm guard column. Hydrolysate 1 was prepared with a lower sulfuric acid concentration (0.5 %) and incubated at a lower temperature (158 °C) causing less sugar degradation. Therefore, it shows a lower 5-HMF (peak 1) and a lower furfural (peak 2) concentration compared to the other two hydrolysates which were prepared with a higher acid concentration (1 % and 1.5 %, respectively) and a higher temperature (180 °C and 190 °C, respectively). As illustrated in Fig. 4b, the 5-HMF analysis results with the 50 mm guard column compared to the 300 mm column were lower for hydrolysate 1 (88.2 %, 0.15 vs. 0.17 mg/mL), lower for hydrolysate 2 (93.6 %, 1.31 vs 1.40 mg/mL) and lower for hydrolysate 3 (84.1 %, 0.74 vs. 0.88 mg/mL). However, the furfural amounts determined with the 50 mm guard column were in the range of 96.8 %-103.8 % of the 300 mm column results (1.49 vs. 1.54 mg/mL, 1.81 vs. 1.87 mg/mL, and 2.46 vs. 2.37 mg/mL, respectively) and can be considered very comparable. In conclusion, the different pretreatment severity profiles were adequately reflected by analysis with the 50 mm guard column.

### Application to analysis of ethanol production and xylose and glucose consumption during fermentations

**Fig. 5:**
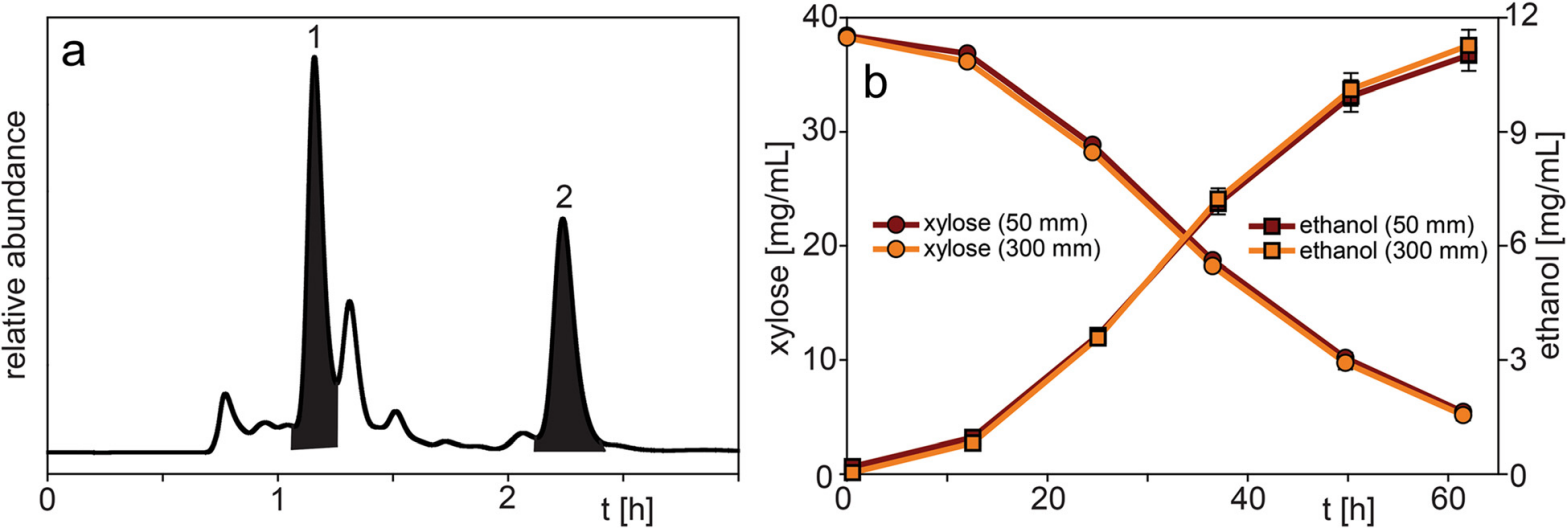
Representative chromatogram of the supernatant from a *Saccharomyces cerevisiae* SA-1-X123 strain fermentation analyzed with (**a**) the 50 mm guard column showing xylose (peak 1) and ethanol (peak 2) elution, and (**b**) xylose and ethanol concentration profile of the supernatant from a modified *S. cerevisiae* strain fermentation between 0 h and 62 h analyzed with the 50 mm guard column and 300 mm column. HPLC parameters: column temperature 55 °C, mobile phase 5 mM sulfuric acid, flow rate 1.0 mL/min (0.6 mL/min for 300 mm column), refractive index detection

**Fig. 6:**
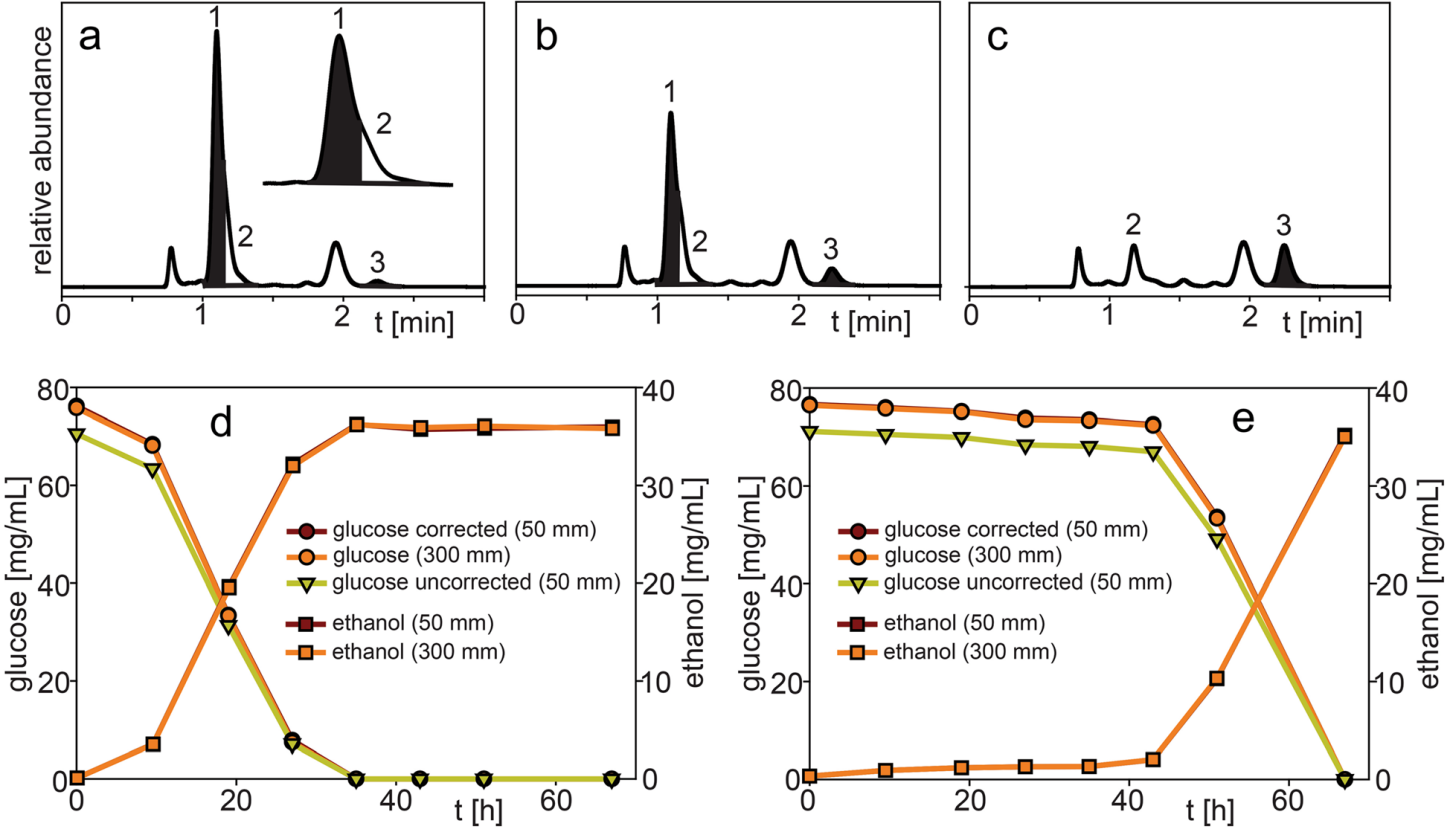
Chromatogram of the supernatant from *Saccharomyces cerevisiae* SA-1 strain fermentation supplemented with 25 % of pH adjusted hydrolysate 3 analyzed with the 50 mm guard column showing glucose (peak 1), xylose (peak 2) and ethanol (peak 3) elution after (**a**) 42 h, (**b**) 50 h and (**c**) 67 h, and xylose and ethanol concentration profile of the supernatant between 0 h and 62 h from a *S. cerevisiae* SA-1 strain fermentation supplemented with (**d**) 25 % of pervaporation detoxified hydrolysate 3 and (**e**) 25 % of pH adjusted hydrolysate 3, analyzed with the 50 mm guard column and 300 mm column. HPLC parameters: column temperature 55 °C, mobile phase 5 mM sulfuric acid, flow rate 1.0 mL/min (0.6 mL/min for 300 mm column), refractive index detection

When glucose and xylose are present in a mixture, their accurate determination using the 50 mm guard column is challenging since the separation of these two sugars is practically not possible. However, the analysis can be performed when either one of the two is present in high abundance or one of the two monosaccharides concentrations is constant over the course of the experiment. Ethanol eluted at 2.2 min which was close to other fermentation media compounds but was still sufficiently isolated for selective measurement in these experiments (Fig. 5a, Fig. 6a-c).

The consumption of xylose and production of ethanol of a modified *Saccharomyces cerevisiae* strain analyzed with the 50 mm guard column and 300 mm column is shown in Fig. 5b and a representative chromatogram is shown in Fig. 5a. The xylose amounts measured with the 50 mm guard column were in the range of 100.5-105.4 % and the ethanol amounts were in the range of 97.7-101.9 % (except for the two lower concentrations) of the values obtained with the 300 mm column. The two lower concentrations of ethanol showed 255.6 % (0.23 vs. 0.09 mg/mL) and 115.6 % (0.89 vs. 0.77 mg/mL) deviation most likely due to interfering co-eluting compounds at these lower concentrations. Overall, the consumption/production profile measured with the 50 mm guard column is very similar to the one obtained with the 300 mm column. However, the total run time using the 50 mm guard column was only 3 min and ethanol elution was completed at 2.5 min. This is about 10 times faster compared to the 300 mm column.

In another experiment, *S. cerevisiae* SA-1 was grown in a medium supplemented with 25 % hydrolysate 3 that was either pH adjusted or detoxified by membrane pervaporation which reduced the concentration of toxic compounds such as furfural, formic and acetic acid [21]. The hydrolysate was obtained by a dilute acid and heat pretreatment of *Miscanthus* biomass in order to release hemicellulosic sugars and improve cellulose digestibility. During this process, the harsh conditions lead to sugar and lignin degradation resulting in the production of a fleet of fermentation inhibitors like furans, phenolics and organic acids which could also interfere with the analysis [22, 23]. Representative chromatograms of the sample with pH-adjusted hydrolysate at time points 42 h, 50 h and 67 h analyzed with the 50 mm guard column are shown in Fig. 6a-c. A decrease of glucose (peak 1) and increase of ethanol (peak 3) can be observed over the time course whereas the remaining chromatogram is mostly the same. The inset in Fig. 6a shows the close elution of glucose (peak 1) and xylose (peak 2) resulting in a peak with a shoulder making accurate integration difficult. Fig. 6d and 6e show the measured glucose and ethanol concentrations over the course of the experiments with the membrane-detoxified hydrolysate and the pH-adjusted hydrolysate, respectively. The values for “glucose uncorrected” were obtained when the attempt was made to integrate glucose separately from the closely-eluting xylose peak (Fig. 6a inset). The determined “glucose uncorrected” concentrations for experiments with the pH-adjusted and with the detoxified hydrolysate were 91.0-93.6 % and 91.6-93.0 %, respectively, of the concentrations obtained when analyzed with the 300 mm column. This indicates that xylose interfered with a correct glucose area determination. The ethanol concentrations were almost identical with 99.4-100.6 % and 99.5-100.2 %, respectively, compared to the results of the 300 mm column. When glucose and xylose were integrated together as one peak and the area of the xylose peak (peak 2, Fig. 6c) of the 67 h time point was subtracted (where glucose concentration was close to 0), “glucose corrected” values were obtained which were closer to the results of the 300 mm column (94.8-100.6 % and 99.5-99.9 %, respectively). Even without glucose correction, the effect of the toxic hydrolysate on delaying the glucose fermentation and production of ethanol until around 42 h into the fermentation was clearly observed with the 50 mm column (Fig. 6e).

### Application to analysis of glucose release from enzymatic digestibility assays of pretreated biomass

**Fig. 7:**
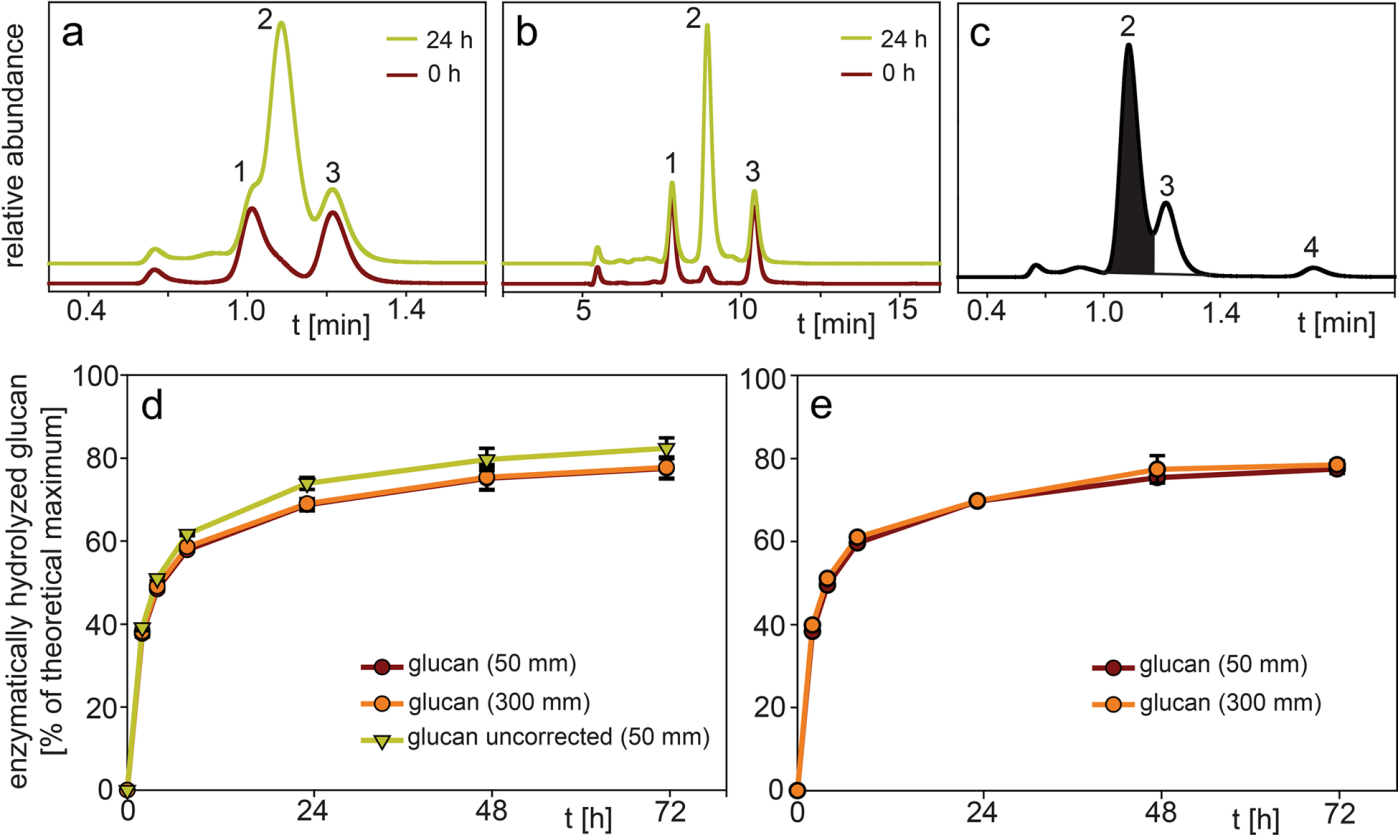
Chromatogram of the supernatant from the enzymatic digestion of “pretreated *Miscanthus* biomass” showing citric acid (peak 1), glucose (peak 2), sorbitol (peak 3) and acetic acid (peak 4) elution after (**a**) 0 h and 24 h digestion in citrate buffer analyzed with the 50 mm guard column, (**b**) 0 h and 24 h digestion in citrate buffer analyzed with the 300 mm column, (**c**) 24 h digestion in acetate buffer analyzed with the 50 mm guard column; and enzymatically released glucan concentration profile of the supernatant from the enzymatic digestion of “pretreated *Miscanthus* biomass”between 0 h and 72 h using (**d**) citrate buffer (**e**) acetate buffer analyzed with the 50 mm guard column and 300 mm column. HPLC parameters: column temperature 55 °C, mobile phase 5 mM sulfuric acid, flow rate 1.0 mL/min (0.6 mL/min for 300 mm column), refractive index detection. Error bars represent +/− 1 standard deviation (n = 3)

The enzymatic release of glucose from cellulose after biomass pretreatment is often performed not only to determine of the effectiveness of the pretreatment itself but also for the evaluation of the hydrolytic potential of new enzymes. A commonly used method is the pretreatment of biomass with dilute acid (e.g. sulfuric acid) at higher temperatures (140–200 °C) which leaves behind a solid cake consisting of mainly lignin and decrystallized cellulose [19, 24, 25]. After washing and/or neutralization this cake is incubated with cellulose-degrading enzymes in either citrate or acetate buffer at pH 5 in order to release glucose [26]. As shown in the chromatogram of the supernatant after enzymatic digestion of “pretreated *Miscanthus* biomass” (Fig. 7a), citrate/citric acid (peak 1) of the citrate buffer is interfering with glucose (peak 2) at lower and higher concentrations on the 50 mm guard column. Furthermore, sorbitol (peak 3) derived from the commercial enzyme mixture (peak 3) also interferes, but to a much lower extent. Using the 300 mm column, citric acid (peak 1), glucose (peak 2) and sorbitol (peak 3) can be separated (Fig. 7b). As observed earlier with the glucose/xylose integration (Fig. 6), the attempt to integrate the glucose peak separately (now without the citric acid peak contribution) lead to glucan conversions (“glucan uncorrected”) that deviated by 2.7-7.1 % from the ones obtained using the 300 mm column (Fig. 7d). For example, glucan conversion after 72 h was 82.4 % (50 mm guard column) vs 77.8 % (300 mm column). At time point 0 h (Fig. 7a), the observed glucose peak (peak 2) was derived from the enzyme mixture used in the experiment. Therefore, this area had to be subtracted as a blank from the glucose area of following time points. Similar as before (Fig. 6e), if the citric acid and glucose peaks at timepoint 0 h were integrated as one peak and subtracted from following peaks (citric acid + glucose) the values obtained with the 50 mm guard column (“glucan corrected”) were in the range of 98.7-99.7 % of the values with the 300 mm column (Fig. 7d). The glucan conversion after 72 h was 77.6 % (50 mm guard column) vs 77.8 % (300 mm column). The elution on the 50 mm guard column is completed after 1.4 min and is therefore about 9 times faster compared to 12.5 min with the 300 mm column.

Using acetate buffer, only sorbitol (peak 3) derived from the commercial enzyme mixture interfered with glucose on the 50 mm guard column (Fig. 7c) because acetate (acetic acid, peak 4) eluted later at 1.75 min. A “split-peak” integration of glucose (peak 2) and sorbitol (peak 3) is sufficient to obtain glucan conversions within 96.2-99.8 % of the ones resulting from using the 300 mm column. The 72 h conversion was 77.5 % (50 mm guard column) vs 78.5 % (300 mm column) and the run time was again about 9 times faster (1.8 min vs 16.5 min) (Fig. 7e).

It is noteworthy to mention that the acetate buffered samples can also be run with an analysis time of only 1.4 min as the citrate buffered samples. The acetate peak (peak 4) will then be eluted within the first 0.4 min of the next sample (not interfering with the glucose peak).

Furthermore, the sample analysis time of 1.4 min is fast enough to compete with other higher-throughput glucose methods like using a YSI bioanalyzer for glucose determination. With the “overlap injection” function of the autosampler, which allows rapid injection of the next sample after a run is completed, about 1000 samples (> two 384-well plates) can be processed per day.

### Application to analysis of ethanol, acetone and n-butanol production during acetone-butanol-ethanol (ABE) fermentation

**Fig. 8:**
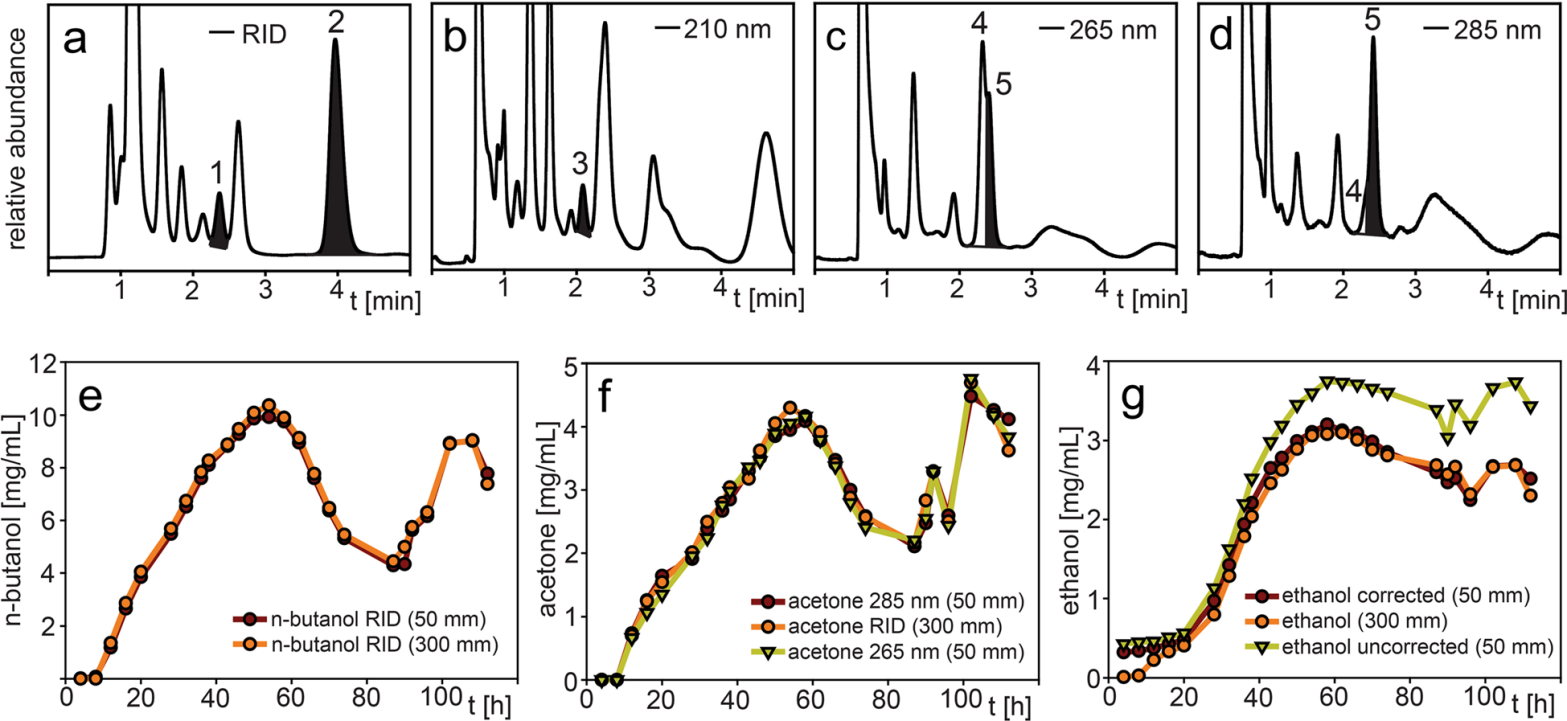
Chromatogram of the supernatant from a *Clostridium acetobutylicum* ABE fermentation after 23 h analyzed with the 50 mm guard column showing ethanol (peak 1), n-butanol (peak 2), ethanol interference (peak 3), acetone interference (peak 4) and acetone (peak 5) analyzed with (**a**) refractive index detection (**b**) UV 210 nm detection (**c**) UV 265 nm detection, (**d**) UV 285 nm detection, and concentration profile of the supernatant from a *Chlostridium acetobutylicum* ABE fermentation of (**e**) n-butanol, (**f**) acetone, (**g**) ethanol, between 0 h and 112 h analyzed with the 50 mm guard column and 300 mm column. HPLC parameters: column temperature 30 °C, mobile phase 5 mM sulfuric acid, flow rate 1.0 mL/min (0.6 mL/min for 300 mm column), refractive index and UV (210 nm, 265 nm, 285 nm) detection

The analysis of the products from acetone-butanol-ethanol (ABE) fermentation with a short column is challenging since *Clostridia* secret a fleet of other compounds (e.g., butyric acid, lactic acid) which can co-elute with the target analytes [8]. The use of an additional UV detector is beneficial for more accurate compound detection and quantification. Fig. 8a shows the chromatogram of a representative sample with RI detection. Ethanol (peak 1) and n-butanol (peak 2) have to be analyzed using the unspecific RI detector. For n-butanol, the concentration profile obtained with the 50 mm guard column is very similar to the 300 mm column (Fig. 8d), since interfering compounds were absent or in low concentration at this retention time. The measured n-butanol concentrations were on average 97.0 % +/− 3.8 % (range 98.1-100.3 %) of the values obtained with the 300 mm column, except for early time points (<16 h) due to lower concentrations affecting accuracy, and the 90 h and 112 h time points with 86.8 % and 105.3 % deviation.

In the 210 nm UV trace of the same sample (Fig. 8b) a compound (peak 3) is visible that is interfering with the ethanol determination using RI detection (note that in the UV trace a compound had a slightly earlier retention time since the RI detector is in line after the UV detector). However, with the 300 mm column, peak 3 and ethanol can be separated (data not shown). If ethanol was determined only by RI detection, concentrations were overestimated and the values ranged 117.5-149.3 % (average 126.9 % +/− 8.4 %) of the values obtained with the 300 mm column (Fig. 8g). The interfering compound was present early in the fermentation process when ethanol production was zero or very minimal. Therefore, it should be possible to measure the area of peak 3 with 210 nm UV detection and correlate it to the area obtained with RI detection. In this way, the interference of peak 3 in RID mode should be reduced by measuring the sum of the area (ethanol + peak 3) with RID and subtracting the theoretical “RID area contribution” of peak 3 calculated from its 210 nm UV area. However, we noticed that the low intensity of peak 3 in the earlier time points made correlation of UV and RID signal less accurate. We therefore used an empirical method to determine the UV/RID correlation by adjusting the correlation factor so the corrected ethanol concentration from the 50 mm guard column best matched the 300 mm column results. This correction factor was applied to the UV-to-RID conversion of peak 3 at all time-points of the fermentation. “Ethanol corrected” values of average 102.8 % +/− 4.8 % (range 94.6-110.8 %) of the 300 mm values were obtained (Fig. 8g), excluding the initial 4–20 h timepoints.

Acetone (peak 5) is more selectively analyzed at a wavelength of 265 nm (absorbance maximum) but in the ABE fermentation sample an interfering peak (peak 4) was present (Fig. 8c). By applying a higher wavelength (285 nm) the area of the interfering peak was greatly reduced (Fig. 8d). Interestingly, despite the interference, even at 265 nm good analysis data (average 96.7 % +/− 5.1 %, range 85.5-105.8 % of the values using the 300 mm column, Fig. 8f) was obtained. The results from the 285 nm detection were similar (average 99.2 % +/− 5.6 %, range 87.3-106.5 % of the value using the 300 mm column, Fig. 8f), excluding the 112 h time point (113.6 %).

The increase and decrease of the profiles of ethanol, acetone and n-butanol during the course of the fermentation was caused by an applied membrane pervaporation which removed these components from the fermentation medium. During the first 50 h, the microbial production outpaced the membrane removal until the accumulated solvents became toxic to the microorganism and stopped further secretion. At this point, a decline in concentration was observed due to removal by membrane pervaporation (50–90 h). Since the concentration of the solvents was eventually reduced to a sub-toxic level, the cells resumed production of acetone, n-butanol and ethanol later in the experiment (90–112 h). Overall, analysis on the 50 mm guard column was able to monitor these changes with sufficient accuracy and with a sample analysis time of only 5 min compared to about 45 min on the 300 mm column.

### Application of the Bio-Rad Micro Guard Cation H guard column

**Fig. 9:**
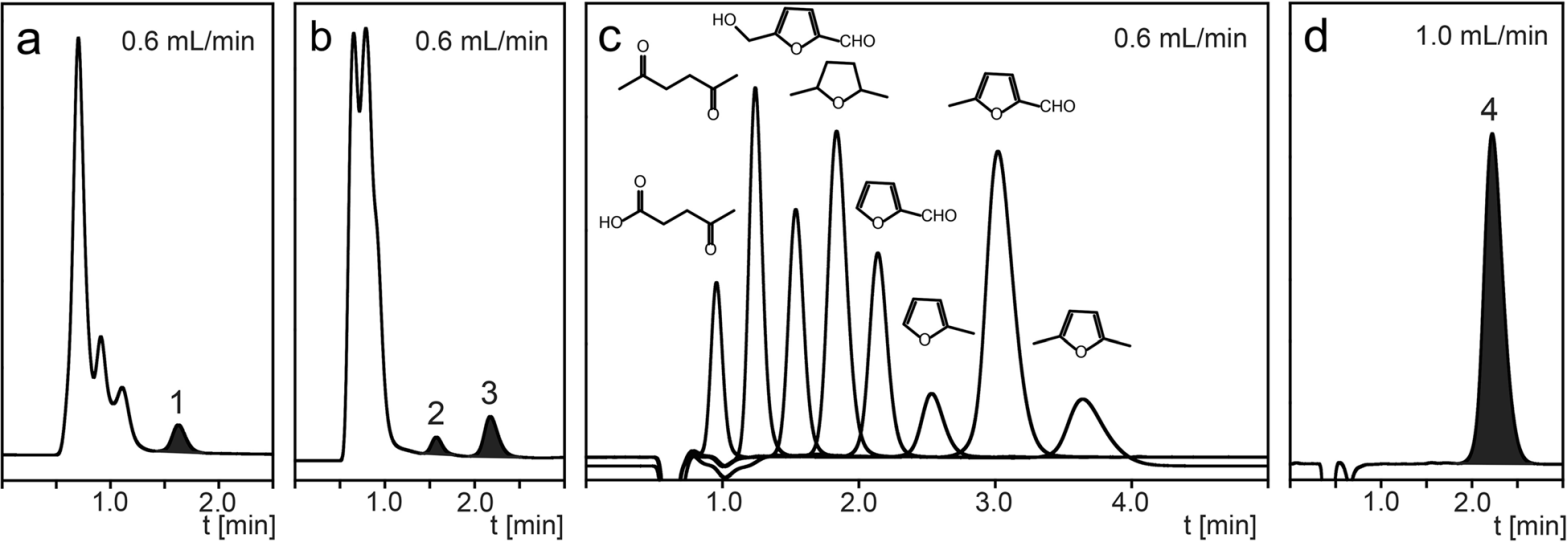
Chromatogram of (**a**) the supernatant of *Escherichia coli* strain DH1 fermentation showing n-butanol (peak 1) elution at 0.6 ml/min flow rate, (**b**) the hydrolysate 3 showing 5-HMF (peak 2) and furfural (peak 3) elution at 0.6 ml/min flow rate, (**c**) a mixture of levulinic acid, 2,5-hexanedione, 5-HMF, 2,5-dimethyltetrahydrofuran, furfural, methylfuran, 5-methylfurfural and 2,5-dimethylfuran at 0.6 ml/min flow rate, (**d**) a standard of n-hexanol (peak 4) at 1.0 ml/min flow rate, using the 30 mm guard column. HPLC parameters: column temperature 55 °C, mobile phase 5 mM sulfuric acid, flow rate 0.6 mL/min (1.0 mL/min for n-hexanol), refractive index detection

An even shorter guard column is the 30 mm x 4.6 mm i.d. Bio-Rad Micro Guard Cation H (“30 mm guard column”). It can be especially well used for the analysis of compounds that elute late on the 300 mm column. For example, Fig. 9a shows its application to the n-butanol analysis mentioned earlier. The analysis results were very similar to the ones obtained with the 50 mm guard column with results ranging 92.0-101.8 % for DH1, 98.2-101.0 % for DH1Δ*acrB* and 88.8-91.6 % for BW25113 profile analysis compared to the 300 mm column (Fig. 2b). However, by using a standard 0.6 mL/min flow rate, the analysis time was cut in half (2 min) compared to the 50 mm column (4 min).

Applied to the analysis of 5-HMF and furfural (Fig. 9b), the analysis time (2.5-3 min) was further reduced by more than half compared to the 50 mm guard column (6–6.5 min, Fig. 4a). Furfural concentrations were slightly higher compared to the 300 mm column: 100.6 % (1.55 vs 1.54 mg/mL), 102.7 % (1.93 vs 1.88 mg/mL) and 109.7 % (2.60 vs 2.37 mg/mL) for hydrolysate 1–3, respectively (Fig. 4b). The concentration for 5-HMF was lower for hydrolysate 1 (64.7 %, 0.11 vs 0.17 mg/mL) and hydrolysate 3 (78.4 %, 0.69 vs 0.88 mg/mL) but identical for hydrolysate 2 (1.40 mg/mL).

An interesting application of this guard column is the analysis of some promising liquid fuel molecules and their precursors [3, 27]. Most of these types of molecules are usually analyzed by gas chromatography. If analyzed by liquid chromatography on the 300 mm column, the mobile phase has to be modified with a higher concentration of an organic solvent, e.g. acetonitrile, in order to reduce their long retention time [27]. Instead of changing the mobile phase composition, we have explored the reduction of the retention time by using the 30 mm guard column. Fig. 9c shows the nearly baseline separation of a standard mixture of levulinic acid, 2,5-hexanedione, 5-HMF, 2,5-dimethyltetrahydrofuran, furfural, methylfuran, 5-methylfurfural and 2,5-dimethylfuran, eluting within only 4 min. Since the latter three compounds elute after furfural their elution times are in the range of 1–2 h on the 300 mm column (data not shown). Interestingly, despite the structural similarity, especially of the furans, the 30 mm guard column provided sufficient separation efficiency for these molecules. When the flow rate on the 30 mm guard column was increased to 1 mL/min a further reduction of the analysis time was achieved. At this flow rate, other compounds eluting within the first 25 min on the 300 mm column now eluted in less than 1 min on the 30 mm guard column (data not shown). For example, the elution of n-hexanol was at 2.3 min at a flow rate of 1 mL/min (Fig. 9d) compared to 96 min on the standard 300 mm column (data not shown). In general, the 30 mm guard column operated at 1.0 ml/min elutes compounds about 4 times faster than the 50 mm guard column (data not shown). Examples are, e.g., 2,5-dimethylfuran (2.3 vs 10.2 min), n-hexanol (2.2 vs 9.3 min) and 2,5-dimethyltetrahydrofuran (1.1 vs 4.3 min). The 30 mm guard column can therefore be an ideal tool for the rapid analysis of selected late-eluting compounds. We therefore encourage researchers to explore this guard column for the analysis of compounds that seem to be “invisible” to the RID when injected on the 300 mm column. This invisibility is most likely due to the effect that compounds remain on the column for a long time and are eluted as broad peaks appearing more like a “baseline-drift”, which makes peak detection and compound quantification difficult or impossible.

## Conclusion

We have used examples from various experiments to show that a 50 mm guard column can be successfully used for the analysis of samples from many bioenergy- and biotechnology-relevant experimental setups. With this column, samples can be analyzed up to ten-times faster compared to the standard 300 mm column with very comparable results. Therefore, it is an ideal tool for processing a large amount of samples, such as in screening or discovery experiments. By applying correct peak integration and interference subtraction, concentration profiles from even more complex samples are reliably obtained. A further decrease of the analysis time was achieved by using a 30 mm guard column that has been shown to be especially suitable for the rapid analysis of compounds with long elution times on the standard 300 mm column, including biofuel-related alcohols (e.g. n-butanol, n-hexanol) and furan- and tetrahydrofuran-type molecules. Owing to the universal applicability and the ease of use as well the numerous citations in the literature, we suspect that almost all labs involved in the fields of biotechnology or biomass conversion technology are equipped with an HPLC-UV/RID instrument and should therefore be able to easily apply the methods presented here. Besides the faster analysis time, guard columns only cost a fraction of their counterparts they were designed to protect. We therefore encourage researchers to explore the feasibility of applying the separation on a guard column to their experimental setup in order to significantly reduce analysis time.

## Methods

### HPLC analysis

Samples were either filtered (0.45 μm) or centrifuged (10 min, 14,000 g) and analyzed using a 1200 series high-pressure liquid chromatography system (Agilent Technologies, Santa Clara, CA, USA) consisting of an autosampler with tray cooling, binary pump, degasser, thermostated column compartment, diode array detector (DAD) and refractive index detector (RI) connected in series. The supernatant was injected onto either a 300 mm × 7.8 mm (length × inner diameter) Aminex HPX-87H (Bio-Rad, Richmond, CA, USA) column with 9 μm particle size, 8 % cross-linkage equipped with a 30 × 4.6 mm micro-guard Cation H guard column cartridge (Bio-Rad, Richmond, CA) or onto a 50 × 7.8 mm Rezex™ RFQ-Fast Acid H^+^ guard column (Phenomenex, Torrance, CA, USA) with 8 μm particle size, 8 % cross-linkage or onto a 30 × 4.6 mm micro-guard Cation H guard column cartridge (Bio Rad, Richmond, CA, USA). Compounds were eluted either at 30, 55 or 80 °C at a flow rate of either 0.6 or 1.0 mL using a mobile phase of 5 mM sulfuric acid.

### n-butanol fermentation

DH1, DH1Δa*crB* and BW25113 *E. coli* strains were inoculated at an OD600 of 0.35-0.45 in 250 mL screw-capped baffled Erlenmeyer flasks in TB (terrific broth) medium (1 L contains 12 g casein peptone, 24 g yeast extract, 4 mL glycerol, 2.2 g potassium phosphate monobasic, 9.4 g potassium phosphate dibasic, Fisher Scientific, Pittburgh, PA, USA) and grown anaerobically at 30 °C and 215 rpm for up to 72 h. DH1Δ*acrB* strain was constructed by removing the efflux pump subunit acrB from the chromosome according to established procedures [28].

### iso-butanol fermentation

sFAB5441 is *E. coli* Dh5αZ1 strain (with LacI and TetR on chromosome) expressing iso-butanol pathway genes. The plasmids for these pathways (pSA55 and pSA69) were obtained from James Liao [29]. Strain sFAB5692 has the same 2 plasmid as sFAB5441 but the genetic background of the strain is AL329 with several deletions (adhE; frdB/frdC; fnr; IdhA; ptA; pflB, adhP, eutG, yiaY, yjgB, betA, fucO, eutE) to improve pyruvate production and iso-butanol yield [30].

All strains were grown aerobically in shake flasks in LB medium and then inoculated in M9 media containing 5 g/L yeast extract, ~50 g/L glucose, and 1000-fold dilution of A5 trace metal mix (2.86 g H_3_BO_3_, 1.81 g MnCl_2_x4H_2_O, 0.222 g ZnSO_4_x7H_2_O, 0.39 g Na_2_MoO_4_x2H_2_O, 0.079 g CuSO_4_x5H_2_O, 49.4 mg Co(NO_3_)2x6H_2_O per liter water) at 37 °C and grown to an OD600 of 0.6-0.8. Cells were then induced with isopropyl-β-D-thiogalactopyranoside (IPTG) and then grown at 30 °C [29].

### Pretreated biomass and hydrolysates

A two-step dilute acid pilot plant pretreatment of *Miscanthus* X *giganteus* (around 1 inch size) was performed in a pilot plant at Andritz, Glens Falls, NY. In the first step, the biomass was heat-pretreated with 0.5 % (w/w) sulfuric acid at 12.5 % (w/w) solid loading applying a rapid steam-driven heating ramp to 158 °C and a holding time of 20 min. The liquid was removed from the solids/pretreated biomass by squeezing out in a hydraulic press and was referred to as “hydrolysate 1”. In the second step, the pretreated biomass was again heat-pretreated with 1 % (w/w) sulfuric acid at 12.5 % (w/w) solid loading applying a rapid steam-driven heating ramp to 180 °C and a holding time of 4 min. The pressed out liquid from this second stage was referred to as “hydrolysate 2”.

Another pretreatment was performed in a pilot plant of the National Renewable Energy Lab (NREL), Golden, CO. *Miscanthus* X *giganteus* (around 1 inch size) was heat-pretreated with 1.5 % (w/w) sulfuric acid at 25 % (w/w) solid loading applying a rapid steam-driven heating ramp to 190 °C and a holding time of 1 min and subsequent rapid pressure release. The pretreated biomass was separated from the mixture by centrifugation and referred to as “pretreated *Miscanthus* biomass”. The obtained liquid phase was referred to as “hydrolysate 3”.

### Xylose to ethanol fermentation

*Saccharomyces cerevisiae* SA-1-X123 (Brazilian industrial strain) including the X123 cassette for xylose fermentation was grown from an OD600 of 1 in yeast-peptone-xylose (YPX) medium containing 40 g/L xylose aerobically at 30 °C and 100 rpm for 62 h [31, 32].

### Glucose to ethanol fermentation

“Hydrolysate 3” was either adjusted to pH 5.5 with KOH, then centrifuged and filter sterilized or detoxified by 24 h pervaporation as described previously [21]. *Saccharomyces cerevisiae* SA-1 was provided by the Yeast Biochemistry and Technology Laboratory, Biological Science Department, Luiz de Queiroz College of Agriculture, University of Sao Paulo, Brazil and was grown at 30 °C at 200 rpm in 10 mL of synthetic complete media (SC-80). SC-80 contains 80 g/L glucose, 2 g/L dropout mix (US Biological, Salem, MA, USA), 6.7 g/L yeast nitrogen base (Becton Dickinson, Franklin Lakes, NJ, USA), 19.5 g/L 2-ethanesulfonic acid (MES) buffer, and a small amount of KOH to adjust the pH to 5.5. After overnight growth, cells were harvested by centrifugation. Fermentation was performed in 25 mL Hungate bottles under anaerobic conditions. The fermentation broth contained either 25 % (v/v) of “hydrolysate 3” or pervaporation-detoxified “hydrolysate 3” (with added water to match the amount removed by pervaporation), with the addition of the components of SC-80 to match SC-80 levels, and harvested SA-1 yeast cells to obtain an initial OD600 of 0.3. The fermentation was performed at 34 °C and 200 rpm for 67 h.

### Enzymatic digestibility assay

“Pretreated *Miscanthus* biomass” was extensively washed with de-ionized water followed by consecutive centrifugation steps until pH of decanted water reached pH 5. Excess water was then removed from the biomass by manually squeezing the biomass between paper towels. An equivalent of biomass containing 1 g of cellulose was used in 25 mL liquid volume digestion reactions (considering water from biomass) in 50 mL Falcon screw cap tubes. Final digestion reactions contained either 0.05 M sodium citrate buffer (pH 4.8) or 0.05 M sodium acetate buffer (pH 4.8) and 30 FPU cellulase from *Trichoderma reesei* ATC 26291 (Sigma-Aldrich, St. Louis, MO, USA), 20 U Novozymes 188 (Sigma Aldrich, St. Louis, MO, USA) and 0.02 % (w/v) sodium azide. The digestion reactions were incubated at 50 °C and 200 rpm for 72 h.

### ABE fermentation and pervaporation

Fermentations were carried out at 37 °C and 200 rpm in 3-L bioreactors (Bioengineering AG, Switzerland) with a 2 L working volume [33, 34]. Seed culture of *Clostridium acetobutylicum* ATCC824 (purchased from the American Type Culture Collection, Manassas, VA, USA) in clostridia growth medium (CGM, 100 mL) was prepared in a 150 mL anaerobic serum bottle at 37 °C until OD600 reached 2.0 and 60 mL of the seed culture was used to inoculate the bioreactor. The bioreactor automatically kept the pH ≥ 5.0 during the fermentation, using a 5 M KOH solution. Nitrogen gas was inserted into the bioreactor at a rate of 200 mL/min to maintain an anaerobic environment. Losses of volatiles, through the gas exhaust port, were minimized by using a cooling condenser attached to a RTE7 water bath (Thermo Fisher-Scientific, Sunnyvale, CA, USA) kept at 4 °C. The system was attached to pervaporation laboratory bench test unit built by Sulzer Chemtech, Neunkirchen, Germany.

After 18 h, pervaporation started with a polystyrene-block-polydimethylsiloxane-block-polystyrene (SDS) block copolymer membrane (37 cm^2^, 2 μm thickness (on support)) [21]. The fermentation broth of the bioreactor was continuously passed over the membrane and back into the bioreactor by using a peristaltic pump (model # 7553–70, Cole-Parmer, Vernon, IL, USA,). ABE fermentation by *C. acetobutylicum* occurs in two steps: an acidogenesis phase wherein the microbes mainly produce acetic acid and butyric acid, followed by a solventogenesis phase wherein the microbes mainly produce ABE.
